# A Palearctic view of a bat fungal disease

**DOI:** 10.1111/cobi.14265

**Published:** 2024-04-15

**Authors:** F. Whiting‐Fawcett, A. S. Blomberg, T. Troitsky, M. B. Meierhofer, K. A. Field, S. J. Puechmaille, T. M. Lilley

**Affiliations:** ^1^ Department of Evolution, Ecology and Behaviour University of Liverpool Liverpool UK; ^2^ BatLab Finland, Finnish Museum of Natural History University of Helsinki Helsinki Finland; ^3^ Department of Biology Bucknell University Lewisburg Pennsylvania USA; ^4^ Institut des Sciences de l’Évolution Montpellier (ISEM) University of Montpellier, CNRS, EPHE, IRD Montpellier France; ^5^ Institut Universitaire de France Paris France

**Keywords:** bats, coevolution, disease management, fungal infection, white‐nose syndrome, wildlife conservation, wildlife disease, coevolución, conservación de vida silvestre, enfermedad de vida silvestre, infección fúngica, manejo de enfermedades, murciélagos, síndrome de nariz blanca

## Abstract

The fungal infection causing white‐nose disease in hibernating bats in North America has resulted in dramatic population declines of affected species, since the introduction of the causative agent *Pseudogymnoascus destructans*. The fungus is native to the Palearctic, where it also infects several bat species, yet rarely causes severe pathology or the death of the host. *Pseudogymnoascus destructans* infects bats during hibernation by invading and digesting the skin tissue, resulting in the disruption of torpor patterns and consequent emaciation. Relations among pathogen, host, and environment are complex, and individuals, populations, and species respond to the fungal pathogen in different ways. For example, the Nearctic *Myotis lucifugus* responds to infection by mounting a robust immune response, leading to immunopathology often contributing to mortality. In contrast, the Palearctic *M. myotis* shows no significant immunological response to infection. This lack of a strong response, resulting from the long coevolution between the hosts and the pathogen in the pathogen's native range, likely contributes to survival in tolerant species. After more than 15 years since the initial introduction of the fungus to North America, some of the affected populations are showing signs of recovery, suggesting that the fungus, hosts, or both are undergoing processes that may eventually lead to coexistence. The suggested or implemented management methods of the disease in North America have encompassed, for example, the use of probiotics and fungicides, vaccinations, and modifying the environmental conditions of the hibernation sites to limit the growth of the pathogen, intensity of infection, or the hosts’ responses to it. Based on current knowledge from Eurasia, policy makers and conservation managers should refrain from disrupting the ongoing evolutionary processes and adopt a holistic approach to managing the epizootic.

## Abstract (es)

Vista paleártica de una enfermedad fúngica de murciélagos

**Resumen:**

La enfermedad fúngica que produce el síndrome de nariz blanca en murciélagos en hibernación en Norte América ha resultado en declinaciones poblacionales dramáticas en las especies afectadas desde la introducción del agente causante, *Pseudogymnoascus destructans*. El hongo es nativo del Paleártico, donde también infecta a varias especies de murciélagos; sin embargo, raramente causa patología severa o la muerte del hospedero. *Pseudogymnoascus destructans* infecta a los murciélagos durante la hibernación invadiendo y digiriendo el tejido de la piel, lo que resulta en la disrupción de los patrones de torpor y la consecuente emaciación. Las relaciones entre el patógeno, el huésped y el ambiente son complejas, y los individuos, las especies y poblaciones responden al patógeno fúngico de distintas maneras. Por ejemplo, *Myotis lucifugus*, especie del Neártico, responde a la infección montando una respuesta inmune robusta, produciendo una inmunopatología que a menudo contribuye a la mortalidad. En contraste, *M. myotis* del Paleártico no presenta respuesta inmunológica significativa a la infección. La falta de una fuerte respuesta, resultado de la larga coevolución entre hospederos y el patógeno en el rango nativo de distribución del patógeno, probablemente contribuye a la supervivencia en especies tolerantes. Después de más de 15 años desde la introducción del hongo en Norte América, algunas de las poblaciones afectadas están mostrando señales recuperación, lo que sugiere que el hongo, hospederos, o ambos, están pasando por procesos que eventualmente pueden conducir a la coexistencia. Los métodos de manejo de la enfermedad sugeridos o implementados en Norte América han abarcado, por ejemplo, el uso de probióticos y fungicidas, vacunaciones y modificación de las condiciones ambientales de los sitios de hibernación para limitar el crecimiento del patógeno, la intensidad de la infección o las respuestas de los hospederos. Con base en conocimiento actual de Eurasia, los formuladores de políticas y los manejadores de la conservación deberían abstenerse de alterar los procesos evolutivos en curso y adoptar un enfoque holístico para gestionar la epizootia.

## A BAT FUNGAL DISEASE

White‐nose disease (WND) is a fungal disease affecting hibernating bats (Blehert et al., 2009), currently considered one of the most detrimental wildlife diseases of modern times (Frick et al., 2016). Since the introduction of its causative agent, the cold‐adapted fungus *Pseudogymnoascus destructans* (= *Geomyces destructans* Blehert & Gargas, 2009 [Gargas et al., 2009]), to North America, the disease, associated with white‐nose syndrome (WNS), has resulted in mass mortality of affected species and has caused unprecedented population collapses in many of the affected areas (Blehert et al., 2009; Frick et al., 2015). Endemic to the Palearctic, the fungus does not cause significant mortality in Eurasian bats, due to extended coevolution between the pathogen and local bat hosts (Drees et al., 2017; Fischer et al., 2020; Fritze & Puechmaille, 2018; Leopardi et al., 2015; Puechmaille, Wibbelt, et al., 2011; Zukal et al., 2014). Presently, after more than 15 years since the introduction of *P. destructans* to North America, some of the affected bat populations have begun showing signs of recovery (Frank et al., 2019), suggesting that the fungus or hosts or both are undergoing processes that can eventually lead to coexistence. We sought to form a synthesis of current understanding of the relationships among host, pathogen, and environment in WND dynamics and provide suggestions on conservation practices for North American bats affected by the disease. Understanding the dynamics of WND in the Palearctic and recognizing the adaptive mechanisms that have allowed species to persist can help predict the fate of Nearctic bat populations. We therefore summarized research from the Palearctic and Nearctic to consider causality with regard to mortality, the definition of disease, how the interactions of the host, pathogen, and environment contribute to disease, the concept of immunological tolerance, and how these could be considered in a holistic approach to viability assessments and planning of conservation measures.

## MECHANISMS OF MORTALITY

Emaciation is considered the ultimate cause of mortality in susceptible Nearctic bat species. Starvation is caused by a disruption to the normal pattern of hibernation. Infected bats arouse from hibernation more frequently and thus deplete fat reserves before the end of the hibernation season (Reeder et al., 2012; Warnecke et al., 2012). Hibernation consists of torpor bouts, where the bat is inactive and its body temperature is close to the ambient hibernacula temperature, and arousals, where body temperature rises and activity resumes (Thomas & Geiser, 1997). During bouts of torpor, bodily functions including metabolism, breathing, blood flow, and the immune system slow down to conserve energy. Occasional arousal is used by bats to rehydrate, defecate, mate, forage when prey are available, or change hibernacula (Blomberg et al., 2021; Boyles et al., 2020). However, arousals are costly, consuming a majority of the fat reserves acquired for the winter (Thomas et al., 1990). Infected North American *Myotis lucifugus*, one of the best‐studied affected species, arouse 3 times more frequently in the final third of the hibernation period than uninfected individuals (Warnecke et al., 2012), which expends large amounts of the fat reserves.

One proposed explanation for the increased arousal frequency seen in susceptible bats is related to fungal damage to patagium (tail and wing membranes). The patagium has an important function during hibernation: it acts as a diffusion membrane for gas exchange to retain homeostasis (Makanya & Mortola, 2007). The initial biotrophic stage of infection by *P. destructans* produces epidermal bundles of fungal hyphae that form cupping structures that are diagnostic of the disease (Meteyer et al., 2009, 2022). The secondary necrotrophic stage is associated with the release of enzymes that digest the dermal layers (Chaturvedi et al., 2010; Meteyer et al., 2022; O'Donoghue et al., 2015; Reynolds & Barton, 2014), and the build‐up of a metabolite, riboflavin, which facilitates deeper tissue invasion (Flieger et al., 2016). This process results in damage to the wing membrane and so disrupts the diffusion process, which causes infected bats to accumulate significant quantities of dissolved carbon dioxide, leading to respiratory acidosis (Warnecke et al., 2013). This forces bats to arouse to hyperventilate, which is one of the proposed mechanisms for torpor disruption (Verant et al., 2014). Hyperventilation also increases water loss via exhalation, and further water loss is caused by the disturbance of fluid regulation in the damaged patagium (Cryan et al., 2010). Evaporative water loss (EWL) is a significant predictor of arousal frequency (Ben‐Hamo et al., 2013; Thomas & Cloutier, 1992; Thomas & Geiser, 1997); susceptible bats may be induced to arouse to rehydrate as well as hyperventilate.

A parallel mechanism for increased arousal is triggered by cytokines, immunomodulatory proteins that are upregulated by the host in response to infection (Antachopoulos & Roilides, 2005). Infection by *P. destructans* induces the production of inflammatory cytokines, such as IL‐6 and IL‐17, during the arousals that take place during hibernation (Field, 2018; Field et al., 2015; Lilley et al., 2019). Irritation, such as pain and itchiness, associated with this inflammation (Riblett et al., 2009), may stimulate the bats to arouse more frequently. With either of the described underlying mechanisms, or both working in tandem, the increased frequency of arousals leads to emaciation and eventual death in the more susceptible bat species. Additionally, the lethal effects of infection may extend beyond the hibernation period with the inflammation leading to immune reconstitution inflammatory syndrome (IRIS) as the host shifts to extended bouts of normothermia in the spring (Meteyer et al., 2012).

## WHITE‐NOSE DISEASE

**FIGURE 1:**
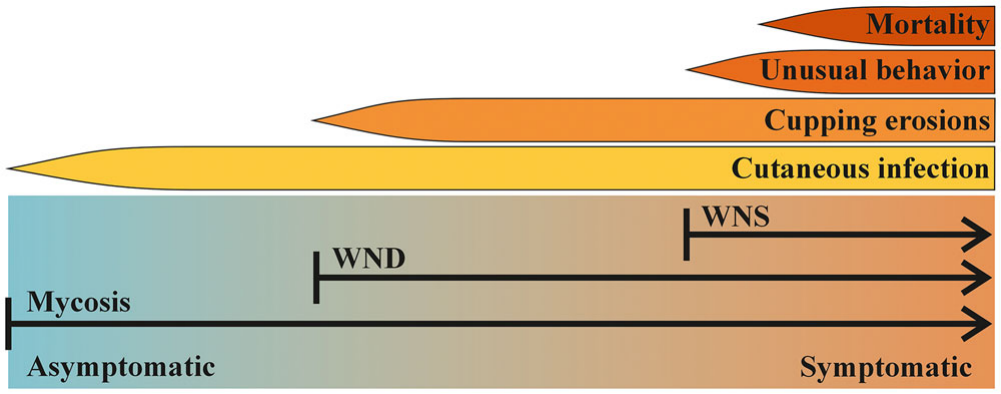
Differences between white‐nose syndrome (WNS), white‐nose disease (WND), and mycosis (axis not proportional to incidence of a given symptom).

The term *white‐nose syndrome* was used in the winter of 2006 and 2007 to characterize the mysterious die‐off affecting hibernating bats in 4 hibernacula in eastern New York (USA), where abnormal behavior was observed (Reeder & Turner, 2008; Veilleux, 2008). However, the use of the term WNS to describe the situation in Europe has led to some confusion (e.g. Chaturvedi & Chaturvedi, 2011), as the conditions there are pathologically similar to those in North America but are not associated with symptoms typically used to characterize WNS, such as increased arousal frequency and emaciation (Fritze & Puechmaille, 2018; Fritze et al., 2021; Pikula et al., 2012; Puechmaille, Wibbelt, et al., 2011; Turner et al., 2014; Wibbelt et al., 2013; Zukal et al., 2016). Using the same terminology to characterize both a disease and a syndrome does not facilitate effective communication regarding the condition, or improve comprehension of the intricate complexities of a system. In medical terminology, a syndrome is “a group of signs and symptoms that occur together and characterize a particular abnormality” (Merriam‐Webster, 2024b), often with multiple or unknown causative agents, whereas a disease is “an impairment of the normal state of the living animal or plant body or one of its parts that interrupts or modifies the performance of the vital functions, is typically manifested by distinguishing signs and symptoms, and is a response to specific infective agents” (Merriam‐Webster, 2024a). With this in mind, and as advocated previously (e.g., Frick et al., 2016), the community should refer to the unusual winter activity and mass mortality of bats as matching the signs and symptoms of WNS and, in contrast, the skin erosions observed as impairment of bat tissue, specifically caused by *P. destructans*, a diagnostic for WND. The presence of the fungus without any signs of the disease is also possible if the pathogen remains superficial, a situation that should simply be qualified as *P. destructans* infection or mycosis (Casadevall & Pirofski, 2000) (Figure 1).

## THE DISEASE TRIANGLE

**FIGURE 2:**
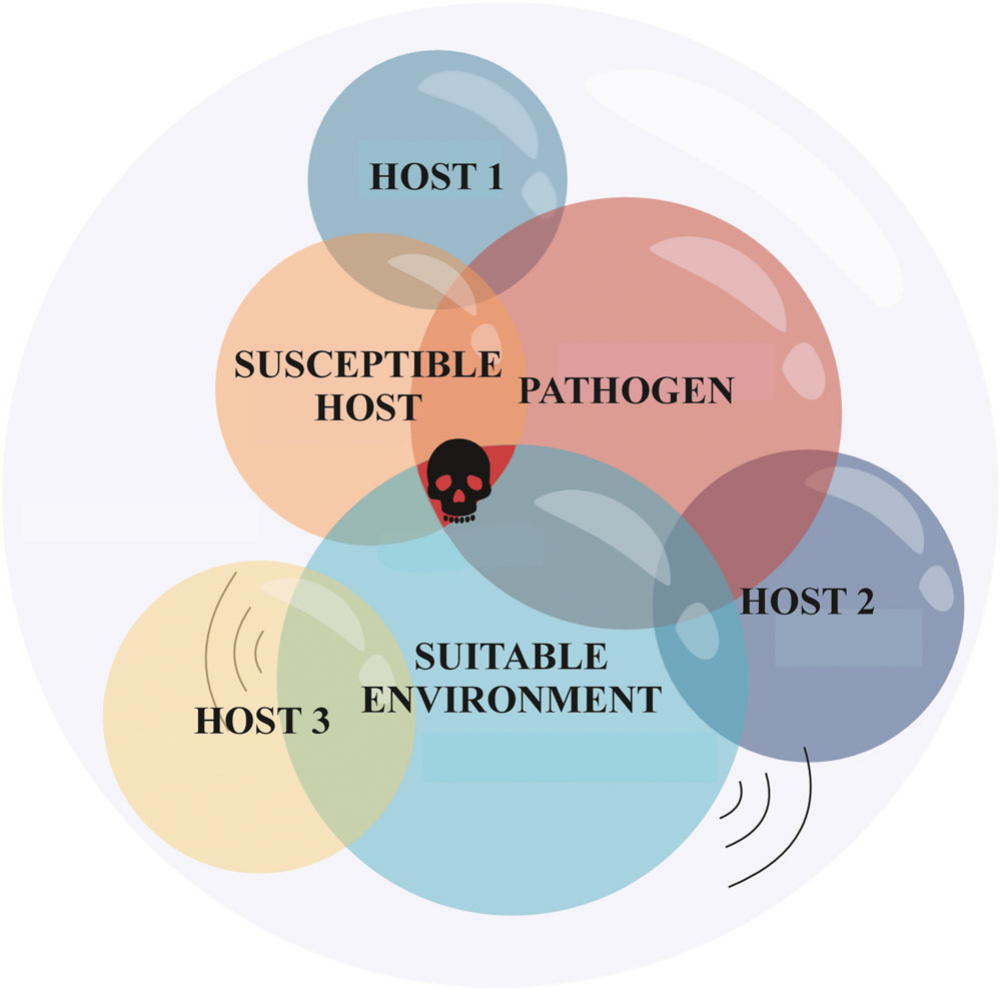
The disease triangle in which disease (skull) occurs at the intersection of the properties of the host, pathogen, and environment (Scholthof, 2007).

Numerous factors, and their interactions, contribute to whether a fungal infection is nonlethal or leads to pathology. To assist in categorizing these factors, they have often been fitted within the disease triangle (Scholthof, 2007), which illustrates disease occurring at the intersection of factors related to the host, the pathogen, and the environment. A shift in any of these factors may lead to manifestation of disease, depending on the direction of the shift (Figure 2). These factors can be abiotic, such as environmental conditions of hibernaculum, and biotic, such as the properties of fungal isolate, the microbiome of either the substrate or the host, hibernation behavior of the hosts, and the inherent susceptibility and immune responses of the hosts to *P. destructans*. We examined current knowledge on WND viewed through the perspective of the disease triangle.

## THE PATHOGEN

*Pseudogymnoascus destructans* is a specialized pathogen of hibernating bats (Minnis & Lindner, 2013), which may have emerged from plant‐associated fungi because it presents invasion strategies similar to fungal pathogens of plants (Meteyer et al., 2022). The coevolution of this fungus with its hosts in the Palaearctic is evidenced by temporal fluctuations in the germination rate of *P. destructans* (peaks during the hibernation [Fischer et al., 2020]); unique enzymatic characteristics of the fungus with reduced saprotrophic enzyme activity; increased activity of enzyme associated with the invasion and digestion of bat skin tissue (Flieger et al., 2016; O'Donoghue et al., 2015; Reynolds & Barton, 2014; Reynolds et al., 2016; Veselská et al., 2020); and the transcription of genes that facilitate the evasion of the host immune system (Reeder et al., 2017). Hibernacula walls are the main environmental reservoir for this pathogen, from which the bat hosts become infected (Fischer et al., 2022). Some evidence of density‐dependent growth on the bats suggests that the fungus may be limited by intraspecific competition (Johnson et al., 2014), which may limit the successful settlement of dispersing *P. destructans* isolates in caves that are already occupied (Fischer et al., 2022). The invasive Nearctic *P. destructans* originated from Europe (Drees et al., 2017; Leopardi et al., 2015), and experimental inoculation suggests that the isolates found in North America are no more pathogenic than its European progenitors (Warnecke et al., 2012). The fungus, which is accumulating mutations, is spreading largely clonally across the Nearctic (Khankhet et al., 2014; Rajkumar et al., 2011; Ren et al., 2012), although signs of recombination have been detected and likely originate from mitotic recombination (Forsythe et al., 2021). Although mutations and changes in growth rate under laboratory conditions have been identified (Forsythe et al., 2018), there is currently no evidence of an increase in pathogenicity being the cause of the disparity in disease outcomes between the Palaearctic and the Nearctic. However, *P. destructans* in the Palearctic shows substantially more diversity than in the Nearctic (Drees et al., 2017; Zhelyazkova et al., 2024). With both heterothallic mating types present in the Palearctic (Dool et al., 2020; Palmer et al., 2014), there is no evidence yet of differences in severity between isolates. Together, these studies of the pathogen suggest that differences in susceptibility to WND between Palearctic and Nearctic species are not primarily driven by changes in the pathogen.

## THE HOSTS

Currently, WND (Figure 1) has been recorded in 12 Nearctic and 31 Palearctic bat species (Hoyt et al., 2021). The species most notably affected by the disease in North America are *Myotis septentrionalis*, *M. lucifugus*, and *Perimyotis subflavus*, which have experienced declines of over 90% in affected hibernacula (Cheng et al., 2021).

Up to 20 bat species in the Nearctic have been recorded with *P. destructans*, many of which share the same underground habitats with the pathogen, but remain asymptomatic (Figure 1). One way that negative infection outcomes may be avoided is to reduce prolonged exposure to the pathogen. For example, bats such as *Lasionycteris noctivagans* and *Lasiurus cinereus* may visit underground sites (Bernard et al., 2015), but they predominantly hibernate in trees (Perry et al., 2010). The presence or activity of the pathogen has not been reported in trees or other similar hibernation sites. Therefore, based on current evidence, these bats are less exposed to the pathogen than strictly cavernicolous species, and their principal hibernation environment is seemingly not suitable for infection to progress and cause damage (Figure 2). Other Nearctic species, such as *Eptesicus fuscus* and *Corynorhinus rafinesquii*, hibernate in underground sites that favor the proliferation of the fungus on the bats (Brack, 2007). However, for some species, such as *E. fuscus*, it is most likely only a minority of the population that hibernate in caves, with the majority of the population overwintering in buildings (e.g., Halsall et al., 2012; Klüg‐Baerwald et al., 2017; Whitaker & Gummer, 1992), rock crevices (Johnson et al., 2017; Lausen & Barclay, 2006; Neubaum et al., 2006), and trees (Zielinski et al., 2007), among other sites (see Agosta [2002] for a review).

While large species, such as the aforementioned, are less susceptible to mortality due to sheer size (Haase et al., 2021), relatively high activity during winter or selecting cold microclimates within the hibernation sites also keeps fungal loads low (Frank et al., 2014; Johnson et al., 2012; Turner et al., 2022). Frequent arousals provide opportunities for the bats to groom off the fungus, inhibiting its proliferation and growth (Brownlee‐Bouboulis & Reeder, 2013). High arousal frequency also enables more opportunities for winter feeding. Foraging and other winter activities are positively correlated with ambient temperature (Avery, 1985; Berková & Zukal, 2010; Blomberg et al., 2021), and therefore populations overwintering in areas with mild winter conditions are likely less susceptible to the disease. However, there may also be species‐specific differences in propensity for winter feeding, facilitating increased arousal frequency and limiting infection in less‐susceptible species (Dunbar et al., 2007; Johnson et al., 2012). In this regard, it appears that species most prone to infection are those that are less likely to feed during the winter (Whitaker & Rissler, 1993) and therefore more dependent on fat reserves gathered prior to hibernation. For instance, depending on fat reserves, the highly susceptible *M. lucifugus* can employ increasingly long torpor bouts during late hibernation, which allows the fungus to proliferate and infiltrate the host tissue effectively (Reeder et al., 2012).

In Europe, the disease is largely associated with *M. myotis* (Puechmaille, Wibbelt, et al., 2011; Wibbelt et al., 2013). *Myotis myotis* has higher pathogen loads and higher prevalence and density of lesions on their patagium than individuals of other species sharing the same hibernacula (Zukal et al., 2014, 2016). Some *M. myotis* individuals have been observed in bouts of torpor lasting over 8 weeks (Blažek et al., 2019), similar to the Nearctic *M. lucifugus* (Jonasson & Willis, 2012), which may exacerbate the fungal load on the host (Fritze et al., 2021). *Myotis myotis* can also have comparable fungal loads to susceptible Nearctic species; studies place this species with either higher (Zukal et al., 2016) or lower (Hoyt et al., 2020) fungal loads than their Nearctic cousins. However, infected *M. myotis* individuals are able to hibernate with marginal to negligible negative effects (Fritze & Puechmaille, 2018; Wibbelt et al., 2013). This ability to host similar pathogen loads, with comparable hibernation behaviors, may indicate an inherent tolerance in *M. myotis* and other infected Palearctic species.

There are several proposed, likely interconnected, mechanisms that promote tolerance to *P. destructans* and limit the fungal load or avoid severe disease outcomes. A large body size is likely protective, providing the host with the energy resources to survive the increased energy requirements of infection. *Myotis myotis*, having one of the highest fungal loads in Europe (Hoyt et al., 2020; Zukal et al., 2016), is also one of the largest species. However, because smaller species (e.g., *M. daubentonii*, *M. dasycneme*, *M. emarginatus*) have similar hibernation patterns to *M. myotis* and do not manifest severe symptoms, additional energy reserves from a larger body size do not appear to be the central factor associated with the tolerance strategy. A strongly supported mechanism is in the immune response of the host itself. Multiple studies point to the susceptible Nearctic *M. lucifugus* mounting a robust immune response to the infection, likely leading to immunopathology that contributes to mortality (Field, 2018; Field et al., 2015; Lilley et al., 2019, 2017). In contrast, *M. myotis* shows no significant transcriptional response to infection (Lilley et al., 2019). Instead, this species appears to use the circulating innate immune effectors without initiating an immunological cascade (Fritze et al., 2021), although recent research suggests the adaptive immune system may be associated with lowered infection intensity in the species (Pikula et al., 2023). This lack of a strong response may be a large contributor to survival in tolerant species (Whiting‐Fawcett et al., 2021).

Falling on the border of the host and the environment, the skin microbiome is also a recently identified pathogen‐limiting defense (Nakatsuji et al., 2021); several microbial taxa have been found with anti‐*P. destructans* properties (Fritze et al., 2012; Grisnik et al., 2020; Hoyt et al., 2015; Lemieux‐Labonté et al., 2020, 2017; Li, Li, Dai, et al., 2022; Li, Li, et al., 2023; Li, Li, Hoyt, et al., 2022). A defensive microbiome can be viewed as part of a holobiont, or a single symbiotic entity in which the host and microbes are mutually dependent on each other for survival (Bordenstein & Theis, 2015; Gilbert et al., 2012; Zilber‐Rosenberg & Rosenberg, 2008). Without the skin microbiome to act as a primary line of defense, a bat in torpor with a downregulated immune system presents a prime opportunity for *P. destructans* to chronically infect the skin of the individual (Casadevall & Pirofski, 2018). Skin‐dwelling symbionts can protect the host by healing wounds (Di Domizio et al., 2020), competing with the pathogen for space and nutrients, or even by directly killing the pathogen (Cogen et al., 2010; O'Neill et al., 2020). Various mutualistic microbes are able to secrete antifungal agents, such as volatile organic compounds (Grice & Segre, 2011), many of which inhibit the growth of *P. destructans* (Cornelison et al., 2014; Micalizzi & Smith, 2020; Padhi et al., 2018). The mere presence of known antifungal taxa found on the skin of bats can inhibit the growth of *P. destructans* both in vitro (Cornelison et al., 2014; Forsythe et al., 2022; Fritze et al., 2012; Grisnik et al., 2020; Hamm et al., 2017; Hoyt et al., 2015) and in vivo (Cheng et al., 2017; Hoyt et al., 2019). Surprisingly, only 7 studies have been conducted on the protective skin microbiome in Palearctic bats so far, leaving a major part of the puzzle contributing to survival unaddressed (Troitsky et al., 2023).

Factors contributing to the susceptibility of a bat species, or a specific entity within a species (e.g., sex; Kailing et al., 2023), to WND are most closely related to hibernation behavior and environmental conditions favored during hibernation. Bats that hibernate for long periods in proximity to *P. destructans* are inherently more susceptible and more likely to develop severe disease outcomes.

## THE ENVIRONMENT

For any disease to manifest, the host and the pathogen must coexist in a suitable environment. Many hibernating bat species and *P. destructans* share an environmental optimum in cool, humid hibernacula, creating appropriate conditions for disease manifestation. Environmental conditions contribute to the disease in a multitude of ways, both directly and indirectly, from the persistence of spores at hibernation sites to the proliferation rate of the fungus (Fischer et al., 2022, 2020; Hoyt et al., 2020; Lorch et al., 2013; Vanderwolf et al., 2016). Also, host infection intensity (Langwig, Frick, et al., 2015) and host identity (Laggan et al., 2023) contribute to the amount of spores shed and therefore to the *P. destructans* load in the environment. Environmental load contributes to whether host populations are stable or experience severe declines (Hoyt et al., 2020).

Environmental conditions largely determine the torpor patterns of bats occupying the hibernacula. Torpor bout duration, which correlates negatively with ambient temperature, plays an important role in WND dynamics. Under laboratory conditions, *P. destructans* grows maximally at temperatures between 12 and 16°C (Verant et al., 2012), and in captivity *M. lucifugus* hibernating at 10°C show higher mortality than those hibernating at 4°C (Johnson et al., 2014). However, the highest fungal loads and probability of WND presence on wild hibernating bats in Europe occur at around 5−7°C (Blomberg et al., 2023; Martínková et al., 2018). This disparity may be due to bats at temperatures closer to the laboratory optimum having too high of a metabolic rate (closely linked to immune system activity; Hotamisligil, 2017) to allow the fungus to proliferate freely. Shorter torpor bouts may also limit fungal growth, as a result of the fungus being groomed off by more frequently arousing hosts (Puechmaille, Frick, et al., 2011). Given that across large geographic scales the mean annual surface temperature of an area correlates with the temperature of hibernation sites, the comprehensive knowledge on the effect of temperature on disease severity has been used to identify high‐risk areas for pathogen introduction and increased monitoring efforts (Blomberg et al., 2023).

Air moisture within a hibernation site is another environmental factor that is simultaneously important for bat hibernation and fungal growth. Environmental air moisture minimizes EWL, reducing dehydration pressure on hibernating bats (Ehlman et al., 2013; Klüg‐Baerwald & Brigham, 2017). Unfortunately for humidity‐loving bat species, *P. destructans* also thrives in high air moisture (Marroquin et al., 2017). That being said, knowledge of the relationships among air moisture levels, disease severity, and bat behavior is limited. Research focused on the susceptible Nearctic *P. subflavus* surprisingly shows no clear impact of humidity on fungal growth (Frick et al., 2022) but does show an indirect effect on female fat loss (McGuire et al., 2021) and notably a general avoidance of the driest hibernacula (85% relative humidity at 8°C) (Boyles et al., 2022). Most studies intending to investigate WND in relation to air moisture have used relative humidity to describe the dryness of the air (e.g., Langwig et al., 2012). However, as demonstrated by Kurta (2014), unless measurements are made at the same temperature (i.e., Marroquin et al., 2017), such values alone do not reliably predict absolute levels of air moisture. Therefore, it is imperative to reassess the findings of these studies with absolute moisture data, which can be derived from raw (unaveraged) relative humidity and temperature data. Regrettably, such data sets are seldom provided by authors, preventing a revisitation of the importance of air moisture on WND. Therefore, approaches incorporating absolute humidity could expand understanding of hibernation and WND.

## MECHANISMS OF SURVIVAL

**FIGURE 3:**
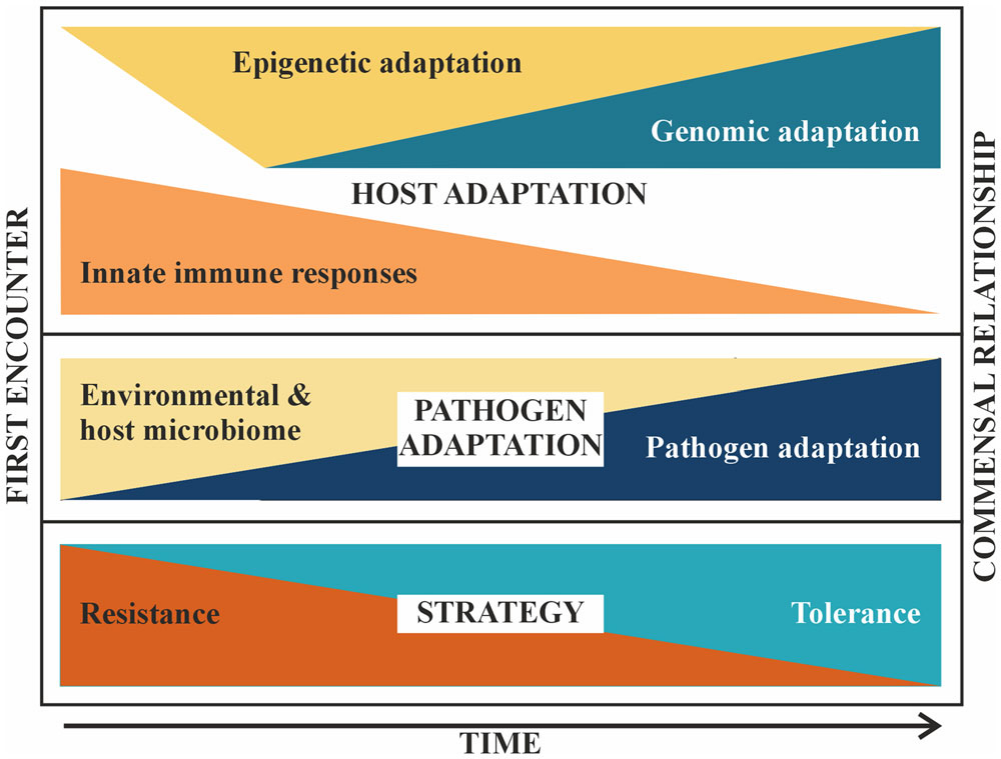
Possible mechanisms contributing to resistance and tolerance and their shifting relevance through time. Figure is for depicting a possible scenario only and is not based on existing data.

Hosts can respond to pathogens with tolerance, resistance, or avoidance (Roy & Kirchner, 2000). Tolerance strategies limit the impact of the pathogen on the host (Medzhitov et al., 2012), whereas resistance mechanisms aim to reduce the negative consequences of the pathogen by limiting the growth of the pathogen (Ayres & Schneider, 2012; Roy & Kirchner, 2000; Schneider & Ayres, 2008). Established models estimate that the maximum fitness of individuals suggests only extreme strategies should evolve: either total resistance or total tolerance (Boots & Bowers, 1999; Boots et al., 2009; Fineblum & Rausher, 1995). Given that tolerance strategies allow the free proliferation of the pathogen, without consequent loss in the fitness of the host, tolerance should be the evolutionarily favored outcome of host–pathogen relationships (Roy & Kirchner, 2000). Because of these estimates, resistance and tolerance are generally considered as alternate, independent strategies (Mazé‐Guilmo et al., 2014). This paradigm could be taken apart by viewing these contending strategies as a continuum where a protective pathway operating through tolerance lies at the far end of host–pathogen responses, with resistance forming the opposing extremity. This would allow a scenario in which mixed strategies are selected in parallel or at different time points within the evolutionary history of a host–pathogen interaction (Figure 3) (Fornoni et al., 2004; Restif & Koella, 2004). Successful resistance, most significantly operating via innate and adaptive immune responses and frequent arousals from torpor, is effective at keeping pathogen loads low, but it may come at a high fitness cost (Mandl et al., 2015). Balancing the fitness trade‐offs between tolerance and resistance can lead the host and pathogen to adapt to the novel interaction and further to coevolve to a commensal relationship (Glass, 2012).

In accordance with these hypotheses, there is indeed wide support for tolerance as the survival mechanism of *M. myotis*, a species with an extended evolutionary history with *P. destructans* (Fritze et al., 2019; Hecht‐Höger et al., 2020). In fact, it appears that *M. myotis* does not elicit a transcriptional response to infection during hibernation (Lilley et al., 2019); it responds only with the already circulating immune effectors (Fritze et al., 2021; but see Pikula et al., 2023). In contrast, the Nearctic *M. lucifugus*, only recently exposed to the pathogen, attempts to control pathogen loads through an immunological resistance strategy, leading to harmful immunopathology (Field et al., 2018; Hecht‐Höger et al., 2020; Langwig et al., 2017; Lilley et al., 2017). More importantly, it appears that bats in the Palearctic have evolved a commensal relationship with *P. destructans* as a result of shared evolutionary history (Davy et al., 2017; Lilley et al., 2019; Zukal et al., 2016). Commensalism can result either from lowered virulence of the pathogen (Lopes & Lionakis, 2022) or from development of tolerance in the host (Atkinson et al., 2013) or both. Although well‐studied examples of fungal pathogens contributing to dampening host responses are known from other study systems (Dalakouras et al., 2023; de Jonge et al., 2012), there is evidence of *P. destructans* attempting to avoid detection by the host immune system in the WND‐susceptible *M. lucifugus* (Reeder et al., 2017). Therefore, although it does not appear to be fully commensal (Casadevall & Pirofski, 2000), the relationship in the Palearctic is an end result of coevolution to which both the host and pathogen contribute.

Although evolution should favor the development of tolerance in host–pathogen relationships, not all Palearctic species that successfully cope with *P. destructans* infection have adopted these strategies. For instance, alongside behavioral defenses, there is some evidence that *Rhinolophus ferrumequinum* may employ resistance to *P. destructans* (Hoyt, Langwig, et al., 2016) (but see Blomberg et al. [2023]) through enhanced immune function at the latter stages of hibernation (Li, Leng, et al., 2023). In addition to resistance, bats may indirectly completely limit *P. destructans* growth by either selecting microclimates that are outside the preferred environmental conditions of the pathogen (Turner et al., 2022) or switching to entirely different hibernacula types, as was hypothesized to be the case for *M. bechsteinii* (Martínková et al., 2010). These different host behaviors add to the complexity of potential host responses as a part of WND dynamics. However, establishing causal connections between infection status and behavioral responses in bats, akin to infection‐induced changes observed in mice (Lopes et al., 2016), has proven challenging.

## TOWARD COEXISTENCE

The first infected populations of Nearctic bats have begun stabilizing (Dobony & Johnson, 2018; Frank et al., 2019; Frick, Cheng, et al., 2017; Reichard et al., 2014), but the mechanisms facilitating persistence are not well understood. Although tolerance should be ultimately favored (Roy & Kirchner, 2000), the Nearctic bat populations may not have had enough time to evolve this strategy, at least at a genomic level (Lilley et al., 2020; but see Gignoux‐Wolfsohn et al. [2021]). However, several changes have been observed in surviving populations. For instance, surviving bat populations appear to be fatter ahead of the hibernation season compared with bats prior to WND emergence (Cheng et al., 2019). This would allow infected bats a higher frequency of arousal from torpor without emaciation. It also appears that highly affected bat species in the Nearctic (*M. lucifugus*, *M. septentrionalis*, and *P. subflavus*) are found at hibernation sites with lower temperatures than were favored by the majority of the populations prior to the epizootic (Johnson et al., 2016; Turner et al., 2022). At least in laboratory conditions, where contributing factors can be isolated, lower temperatures correlate negatively with the growth rate of the fungus (Verant et al., 2012) and facilitate longer torpor bouts for decreased energy expenditure (Thomas et al., 1990). However, this oversimplifies the conditions affecting hibernation at natural sites and requires further insight from field studies. Furthermore, hibernation in large agglomerations is most likely to be one of the main factors contributing to the rapid spread of the disease in the Nearctic (Frick et al., 2015). In fact, the impact of the disease appears to have eliminated the large differences in abundance of bats in Palearctic and Nearctic hibernacula prior to the emergence of the disease. In locations where the disease is manifesting in the Nearctic, a higher fraction of bats appear to be hibernating individually or in smaller agglomerations when compared with pre‐epizootic (Frick et al., 2015; Langwig et al., 2012), but whether this is a behavioral response or purely due to the decline of population size overall is unknown. In comparison, an increase in population size correlates with the number of clusters rather than cluster size in the Palearctic (Martínková et al., 2020).

Infected *M. lucifugus* from surviving populations also show dampened immune responses compared with populations just coming into contact with the pathogen (Lilley et al., 2019). However, at the genomic level, studies show mixed evidence with regard to selection on standing genetic variation that would explain dampening of immune responses (Auteri & Knowles, 2020; Gignoux‐Wolfsohn et al., 2021; Lilley et al., 2020). Epigenetic processes provide a plausible avenue for rapid response after the introduction of a novel pathogen. Epigenetic actions that can modulate the host defense against microbial pathogens include DNA methylation, histone modification, and the activity of noncoding RNAs (Jones, 2012; Laine et al., 2023). Ultimately, alterations of, for example, DNA methylation and the resulting changes in gene expression may generate heritable population‐level phenotypic variation, which can be acted upon by natural selection and drive population adaptation to novel pathogens (Garcia et al., 2019). Whether epigenetic processes are contributing to the dampening of responses, acting either on the transcription of the host or pathogen genes (Garcia et al., 2019; Morandini et al., 2016), has not been investigated in the context of WND.

Genomic adaptation to a new host–pathogen interaction through evolutionary processes, such as selection from standing variation, occurs over several generations, with adaptation through de novo mutations taking even longer (Booker et al., 2017). Therefore, other factors may initially contribute to making the interaction less harmful to the host. For instance, differences in skin microbial communities have been documented between bat populations exposed or not exposed to *P. destructans* (Lemieux‐Labonté et al., 2017), and some microbes inhibit the growth of the fungus (Fritze et al., 2012; Grisnik et al., 2020; Hoyt et al., 2015; Li, Li, Hoyt, et al., 2022; Singh et al., 2018). With an ability to inhibit the growth of a novel pathogen and potential for rapid adaptation, the microbiome can provide a buffer allowing populations to persist until an appropriate evolutionary genomic response has been selected for (i.e., genetic assimilation [Robinson & Pfennig, 2013]). Furthermore, the environment also has a microbiome, which in the case of the environmentally transmissible pathogen, such as *P. destructans*, can have a significant influence.

In the Nearctic, large die‐offs have been reported in only 3 species (*M. lucifugus*, *M. septentrionalis*, and *P. subflavus* [Cheng et al., 2021]). It is important to understand that the overlap in distribution of the host and pathogen is likely greater than the distribution range for the incidence of the disease causing mortality (Xu et al., 2023). Furthermore, high incidence of mortality appears restricted to a proportion of the overlapping distribution range, where factors contributing to disease intercede favorably (Blomberg et al., 2023). For instance, although the distribution range of *M. lucifugus* and *P. destructans* spans longitudinally across the entire continent of North America, large‐scale mortality has been observed mostly in the intensively mined karst regions on the eastern portion of the continent, whereas western populations appear less affected (Udell et al., 2022). Host behavior and environmental conditions favor the generation of an epizootic in these regions affecting much of the population, although disease can occur sporadically elsewhere too when conditions are met.

Although it is difficult to tease apart the relative importance of factors contributing to disease in the Nearctic, examining the properties that Palearctic bats have acquired to face the negative consequences of WND during their evolutionary history may provide insights into what are possible outcomes after extended coexistence (i.e., apparent commensalism [Fritze et al., 2019; Lilley et al., 2019; Twort et al., 2023]). Even in the Palearctic, infection is moderated by variability in the factors present in the disease triangle, leading to varying outcomes even after apparently lengthy exposure times (Hoyt, Sun, et al., 2016; Zukal et al., 2014).

## THE PHILOSOPHY OF DOING NOTHING

Despite populations showing signs of stabilization or recovery, managing the spread and effects of the epizootic is very much an active effort in North America. Action is required by governmental mandate in the United States when threatened or endangered species are involved (U.S. Fish & Wildlife Service, 2022). Therefore, several management strategies have been suggested and trialed to counter the negative consequences of WND (Table 1). To assist in quantifying the management methods tested so far, on 8 August 2023, we performed a Web of Science (WoS) search with the following search terms: ALL = (“*bats*” AND “*management*”) AND ALL = (“*white‐nose syndrome*” OR “*WNS*” OR “*white‐nose disease*” OR “*WND*”).

**TABLE 1:** Publications with tested management methods, including number of papers per method, how the method was tested (model, field, lab), and what the effect was (NA, not available; NE, no effect; Neg, negative; Pos, positive).

	No. of papers	No. of methods used			Total of outcomes			
Management method		Model	Lab	Field	NA	NE	Neg	Pos
Antifungal compounds	9	16	4	3	0	6	7	10
Culling	3	3	0	0	0	0	2	1
Host health	8	7	4	0	1	1	3	6
Host health, antifungal compounds	3	4	0	0	1	0	1	2
Modifying surrounding host summer habitat	2	1	1	1	1	0	0	2
Management of host winter habitat	3	1	0	2	1	1	0	1
Modifying hibernation sites	8	7	2	2	3	3	3	2
No management	1	1	0	0	1	0	0	0

*Note*: For grouping methods, see Appendix S1.

The WoS search resulted in 195 published papers, of which 2 were discarded based on the title. The resulting 193 published peer‐reviewed papers (Appendix S1) were reviewed, and data were collected on whether a treatment was tested, the type of treatment tested, whether it was tested in the laboratory, in the wild, modeled, or a combination of these, and whether the practice had an effect (positive, negative, or none). A positive result does not mean that the treatment cured the disease but rather that the treatment had a significant positive effect on the simulated or measured parameter (e.g., survival, fungal load, use of hibernation site, etc.). Besides, some positive effects are long lasting (e.g., modifying habitat), whereas others are rather short lived (e.g., antifungal compounds). We further excluded 2 methods that occurred in the literature search: monitoring (7 papers) and education on or attitudes toward bats (5 papers) because they do not have an effect that can be easily measured. Out of the remaining 181 papers, 35 tested one or multiple treatments or management practices (treatments in Table 1). Most management methods considered were model based (70.2%), followed by laboratory based (19.3%) and field based (10.5%). Positive results were reported for 6 treatment classes: use of antifungal compounds (10 of 23 tests), culling (1 of 3 tests), improving host health (6 of 11 tests), a combination of antifungal compounds and improving host health (2 of 4 tests), modifying summer habitats (2 of 3 tests), and modifying hibernation sites (2 of 11 tests). In contrast, negative effects were reported for 5 treatment types, including the use of antifungal compounds (7 of 23 tests), culling (2 of 3 tests), improving host health (3 of 11 tests), the combination of antifungal compounds and health improvement (1 of 4 tests), and modifying hibernation sites (3 of 11 tests). In the case of other tests, the researchers reported the management strategy had no effect or the effect on WND could not be concluded. The only management methods with positive or no effect were the modifications of summer and winter habitats.

The efforts so far highlight the importance of pinpointing research gaps to help identify suitable timing of action (Grider et al., 2022; Langwig, Voyles, et al., 2015), management priorities (Bernard et al., 2020), and feasible methods that provide a long‐term solution (Verant & Bernard, 2023). For instance, any individual treatment (e.g., vaccinations and administration of antifungal compounds) will be logistically difficult to administer to a sufficient number of bats on a regular basis, not least because many hibernation sites are yet unknown (Weller et al., 2018) and those that are known may be inaccessible by humans. A treatment or management action also appears to facilitate the recovery of affected populations only if the disease is mild (Fletcher et al., 2020).

Although some management methods, such as the use of probiotics to enhance the protective microbiome against WND, have shown promising results for a single bat species under controlled laboratory settings (Cheng et al., 2017; Hoyt et al., 2015), it is unlikely to significantly decrease the spread of the pathogen and associated disease. Additionally, the effects of probiotic use as a defensive measure can be unpredictable because the topic remains vastly understudied, especially under the large range of natural conditions encountered by hibernating bats. For example, diversity within the cutaneous microbiome is associated with bat species resistant to WND (Vanderwolf et al., 2021), suggesting a consortium of antifungal microbes, instead of just one species or strain, could potentially be an optimal mitigation strategy for WND in the future. However, each microbial taxa introduced to an ecosystem poses a risk because it could potentially act as a pathogen to the other organisms living in the environment, like the often cited *Pseudomonas fluorescens* (Barker et al., 1991; Pompini et al., 2013; Sadd & Schmid‐Hempel, 2006). In some cases, probiotics may even worsen disease severity (Cheng et al., 2017). This is particularly the case when the treatment is occurring before exposure to the pathogen. However, the exact timing of bats being exposed to *P. destructans* has not been identified (Fischer et al., 2022): timing that likely exhibits variation across different species, geographical regions, sexes, age groups, and other factors. Consequently, although modeling outcomes and laboratory experiments may yield encouraging results, achieving scalability in implementing them adequately in the field, perhaps on an annual basis, across extensive areas like North America does not seem feasible in practice.

Management actions also have the potential to keep individuals alive that would not be able to survive in the presence of *P. destructans* and hence to dilute adaptive genetic variation, slowing down the evolution of resistance and tolerance. Considering this, doing nothing, which is a choice that may be driven by unwillingness or inability to act (due to lack of information), and taking preventative rather than interventive strategies (Ashley‐Smith, 2018) presents an alternative angle to disease management. In this scenario, populations left to their own devices are expected to survive as a consequence of natural processes. Of course, not performing management actions is context specific (Bernard et al., 2019; Verant & Bernard, 2023) because it is also critically important that research on the topic does not come to a standstill (Reeder et al., 2016) and include ethical considerations (Ashley‐Smith, 2018). Nevertheless, in accordance with the philosophy of doing nothing, the Nearctic species would likely follow the same pattern as the Palearctic, leading to coexistence through evolution. However, there are some important differences to consider. First, the current epizootic is occurring during the Anthropocene, in which organisms are facing additional threats from rapid environmental change (Pereira et al., 2010), climate change (Blois et al., 2013), energy production (Frick, Baerwald, et al., 2017; Gaultier et al., 2020), and a cocktail of environmental pollutants (Cable et al., 2022). Ongoing is the largest number of extinctions since the last mass extinction (IBPES, 2019): a very different scenario to what bat populations in the Palearctic may have faced during the early stages of their infection history (Leopardi et al., 2015). These additional threats to populations contribute to the formation of extinction vortices, multiplying the probability of eradication (Palomares et al., 2012). Therefore, doing nothing cannot be applied as such, at least without an increased probability of extinction of affected species. However, any intervention demands intellectual (host‐specific knowledge) and manual skills to carry out the task without causing irreversible harm (Ashley‐Smith, 2018).

As research on WND continues, we believe there is enough knowledge to suggest less‐individualistic management methods that alleviate bat stressors, because they will not only promote the health of the bat fauna in entirety, but also promote adaptations allowing the host to persist with the pathogen (Bernard et al., 2020; McCallum, 2012). In other words, rather than targeting a single stressor (*P. destructans*), we advocate for data‐driven management actions that act on key parameters that are generally important for bat (and ecosystem) health (Verant & Bernard, 2023). These include the protection of available hibernacula, minimizing bat disturbance (Thomas, 1995), decreasing environmental contaminants (Bayat et al., 2014; Lilley et al., 2013; Oliveira et al., 2018; Secord et al., 2015; Wu et al., 2020), limiting wind turbine‐related mortality (Erickson et al., 2016; Gaultier et al., 2020), and raising public awareness (Salleh et al., 2020; Shapiro et al., 2021).

Given that limited resources are available, management strategies with a more holistic approach than an individualistic approach may be easier to enact and, crucially, benefit the entire ecosystem rather than potentially harming it (Meierhofer et al., 2022). Implementing these measures will not only yield immediate benefits, but also lead to long‐lasting positive consequences for bats and many other organisms, including humans. Moreover, contrary to the application of antifungal compounds or vaccines, these initiatives have the potential to be self‐sustaining over time. Additionally, species that are more susceptible to WND should be favored in conservation planning: those utilizing extended torpor bouts, preferring higher humidity, hibernating in large aggregations, and expressing immunopathology.

WND is devastating and will continue to affect new bat populations across North American, most severely affecting gregarious, cave‐dwelling bats. However, more recent data show that species have the potential to tolerate or resist this disease. Management strategies require an overall understanding on the intersection of factors contributing to disease to help determine why certain species are more prone to being severely affected. This helps predict the impact on bat populations as WND is documented in uninfected areas, and assists in planning and implementation of effective conservation measures. Furthermore, planning should consider the rapidly changing environment of the Anthropocene. Climate change will have an effect on the distributions of hosts, disease manifestation, and on how populations can recover (Blomberg et al., 2023). With time and carefully planned conservation measures, bats in currently infected populations have the potential to recover, and newly infected populations on the American continent can persist long enough to evolve mechanisms that allow them to resurrect and thrive. Not all species or populations may survive, but the goal should be to ameliorate the evolutionary process taking place during host–pathogen coexistence and to allow *P. destructans* to coevolve with its host from a deadly pathogen into an innocuous endemic.

## Supporting information

Supporting Information
